# Sustainable 3D-Printed Supports Coated with Zirconium-Based Metal–Organic Frameworks for Picolinic Herbicide Extraction

**DOI:** 10.1021/acssuschemeng.6c01944

**Published:** 2026-04-22

**Authors:** Alejandro Gil-Aparicio, Jana Maren Glatz, Jesús Cases Díaz, Enrique Javier Carrasco-Correa, Mónica Giménez-Marqués, José Manuel Herrero-Martínez

**Affiliations:** † Department of Analytical Chemistry, 16781 University of Valencia, Av. Vicent Andrés Estellés, 19, Burjassot, Valencia 46100, Spain; ‡ Instituto de Ciencia Molecular, 201469 University of Valencia, C/Catedrático José Beltrán Martínez, 2, Paterna, Valencia 46980, Spain

**Keywords:** metal−organic frameworks, 3D printing, fused deposition modeling, green UiO-66 synthesis, sustainable fabrication, picolinic herbicides

## Abstract

Metal–organic frameworks (MOFs) offer outstanding potential for pollutant extraction; however, their practical use is often hindered by difficulties in handling and recovery. Herein, we report a sustainable MOF-based extraction platform that integrates 3D-printed supports fabricated from poly­(lactic acid) and pine wood residues with zirconium-based MOFs grown via a green in situ synthesis. Postprinting surface functionalization enabled the stable immobilization of UiO-66-derived MOFs, selected for their strong affinity toward picolinic herbicides (PHs). The resulting UiO-66-OH@3D-printed device demonstrated high extraction efficiencies (73–107%), excellent sensitivity (limits of detection of 0.0016–0.0032 μg L^–1^), and good reusability over eight extraction cycles. The platform was successfully applied to the determination of trace PHs in environmental water samples. This work establishes MOF-functionalized sustainable 3D-printed devices as a robust, cost-effective, and environmentally benign strategy for green water pollutant extraction.

## Graphical abstract

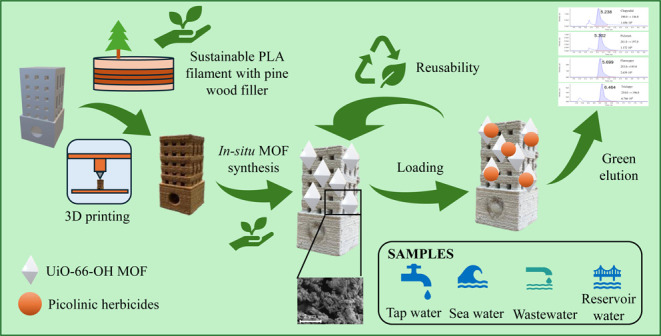

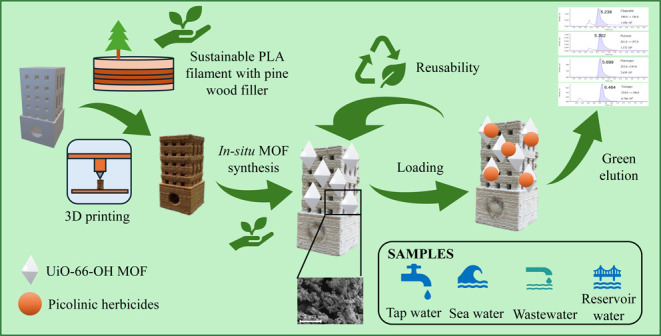

## 1 Introduction

The extensive use of herbicides in modern agriculture has led to their frequent occurrence in surface, ground, and drinking water, raising significant environmental and human health concerns. Many herbicides exhibit high water solubility and chemical stability, which promote their mobility and persistence in aquatic systems and hinder their efficient removal. Consequently, sensitive and selective analytical methods capable of extracting trace-level herbicides from complex aqueous matrices are required to accomplish these tasks with increasingly stringent regulatory limits.

Metal–organic frameworks (MOFs), composed of inorganic ions (metals or metal clusters) and organic molecules (ligands), have emerged as promising candidates for water pollutant extraction owing to their exceptionally high surface area, tunable porosity, and chemically versatile surfaces.^1,2^ These features enable strong and selective interactions with a wide range of organic contaminants.^3^ Despite their excellent adsorption performance, the practical application of MOFs is often hindered by their powdered nature, which complicates handling, recovery, and reuse and limits their integration into analytical workflows. Therefore, the development of strategies that enable the immobilization of MOFs within robust and reusable solid architectures is highly desirable.

Additive manufacturing,^4,5^ particularly fused deposition modeling (FDM), offers a compelling solution by enabling the fabrication of customized, reproducible structures with controlled geometries and mechanical stability. FDM is cost-effective, technically accessible, and compatible with a broad range of thermoplastic filaments.^6^ Although FDM-printed materials have been investigated as extractive phases or as supports for adsorbents,^7,8^ their intrinsically low surface area and limited selectivity restrict their standalone extraction performance. In this context, combining MOFs with FDM-printed supports offers a synergistic approach, integrating the selectivity of MOFs with the structural advantages of 3D printing.

To date, MOF-functionalized FDM-printed devices have been reported mainly for dye removal, while their application to environmentally relevant water pollutants remains scarce.^9–11^ Moreover, existing strategies frequently rely on time-consuming postprinting MOF growth procedures and rarely consider sustainability aspects related to both MOF synthesis and printing materials. In parallel, increasing attention has been devoted to the development of sustainable 3D-printing filaments, such as polylactic acid (PLA) blended with lignocellulosic biowaste, which offer renewable, low-impact alternatives to conventional petroleum-based materials and may act as suitable substrates for green MOF synthesis, thereby enabling the development of fully sustainable MOF-based extraction platforms.^12^

In this work, we address these challenges by developing sustainable MOF-coated 3D-printed supports for water pollutant extraction using four picolinic herbicides (PHs)—picloram, fluroxypyr, triclopyr, and clopyralid—as representative model contaminants (see Table S1 for structures and key physicochemical properties). PHs are widely used agrochemicals characterized by high water solubility and environmental persistence, making their extraction and detection particularly demanding. Among them, herbicides such as clopyralid and picloram exhibit high water solubility (>6 g L^–1^), which contributes to their persistence in aquatic environments and a subsequent health risk for ecosystems and human health.^13,14^ Accordingly, the target pesticides of this study, as well as related pyridinecarboxylic acid derivatives, have been reported in environmental waters at trace concentrations, typically ranging from a few to several 100 ng L^–1^, depending on agricultural application and monitoring location.^15–17^ For instance, fluroxypyr has been detected at concentrations of around 100 ng L^–1^ in water samples collected from different aquatic environments in Spain.^17^ These levels are environmentally relevant because they are close to or above regulatory limits established for drinking water. In the European Union, the maximum admissible concentration for an individual pesticide in drinking water is 0.1 μg L^–1^,^18^ while the US Environmental Protection Agency has established a maximum contaminant level (MCL) of 0.5 mg L^–1^ for picloram,^19^ while no federal MCL values have been set for other herbicides of this family. Liquid chromatography-tandem mass spectrometry (LC-MS/MS) is the most widely used technique for their determination;^15,20–22^ however, trace-level analysis in complex water matrices typically requires an effective sample pretreatment step, as the high polarity and solubility of these compounds often result in low extraction efficiencies and inadequate recoveries. Therefore, the development of efficient and selective extraction materials is required to overcome the limitations commonly found in the analysis of highly polar PHs. In this context, MOFs represent a promising solution due to their high surface area, tunable porosity, and chemically versatile surfaces. Accordingly, several MOF-based sorbents have been reported for the extraction and preconcentration of herbicides, particularly acidic herbicides structurally related to the target analytes of this work, in food and environmental water samples.^23–26^ However, most of these approaches rely on powdered MOF materials either used in dispersive extraction procedures or packed into solid-phase extraction (SPE) cartridges, which may introduce practical limitations in sorbent recovery, additional handling steps, and limited packing reproducibility. In addition, many of the MOFs employed in these studies are synthesized using conventional solvothermal methods that require organic solvents, long reaction times, and energy-intensive conditions, which compromise the overall sustainability of the analytical procedure. These drawbacks highlight the need for alternative strategies that enable greener MOF preparation, together with sorbent formats that are easier to handle, more reusable, and better suited for practical analytical applications.

Herein, we present a simple and efficient postprinting approach for coating 3D-printed devices made from sustainable composite filaments composed of PLA and pine wood residues coated with MOFs. Different frameworks were selected based on their feasible mild and green synthesis, as well as for their high chemical stability in aqueous media,^27^ including UiO-66­(Zr), MOF-801­(Zr), MIL-100­(Fe), and MIL-53­(Al).

Prior to MOF incorporation onto the 3D-printed devices, a preliminary screening study (based on dispersive extraction experiments) was carried out to select the most suitable framework among the tested candidates. As a result of this evaluation, UiO-66 showed the highest retention for the target PHs. This material and its functionalized derivatives obtained with the more hydrophilic amino-1,4-benzenedicarboxylate (NH_2_–BDC) and hydroxy-1,4-benzenedicarboxylate (OH–BDC) were further investigated, taking advantage of the structural versatility of UiO-66-type MOFs. This topology allows the introduction of polar functional groups, which can promote hydrogen-bonding and polar interactions with the target herbicides, improving the retention of highly polar analytes. To facilitate the immobilization of the selected MOFs onto the 3D-printed substrates, the PLA–pine wood scaffolds were first functionalized with maleic anhydride (MA) to introduce reactive surface sites, followed by the in situ growth of zirconium-based MOFs under mild conditions. This study demonstrates a facile, sustainable, and high-performance platform that bridges sustainable additive manufacturing and advanced porous materials for green water pollutant analysis, with novelty arising from the integration of renewable PLA–wood supports, green synthesis of UiO-66-type MOFs, and their application to the extraction of polar herbicides.

## 2 Experimental Section

### 2.1 Materials and Instrumentation and Samples

Details are included in the Supporting Information.

### 2.2 Solvent Compatibility of 3D-Printed Materials

To evaluate the stability of 3D-printed materials in different solvents prior to MOF coating, pieces of 3D-printed material (around 0.7 g) were immersed in different solvents (5 mL) at room temperature (water, methanol (MeOH), ethanol (EtOH), isopropanol (IPA), acetonitrile (ACN), dimethyl sulfoxide (DMSO), dimethylformamide (DMF), NaOH 1 M, NH_3_ 1 M, HCl 1 M, and acetic acid (HAc) 1 M) for a period of 24 h. The mass and volume of the 3D-printed objects were measured after this time. The test was repeated three times to assess reproducibility.

### 2.3 Synthesis of Powdered MOFs

#### 2.3.1 Synthesis of UiO-66’s­(Zr)

The synthesis of UiO-66­(Zr) and derivatives UiO-66-X (X = NH_2_, OH) was performed according to the guidelines previously reported by some of the authors with some modifications.^28,29^ A zirconium oxocluster precursor ([Zr_6_O_4_(OH)_4_(Ac)_12_]_2_·(HAc)_6_) was first synthesized. Briefly, 5 mL of zirconium propoxide (Zr­(OPr)_4_) (11.70 mmol) 70% in 1-propanol was added to 3.75 mL of glacial acetic acid (HAc, 65.25 mmol) in a closed container. The reaction mixture was left at room temperature without stirring for 24 h. A white crystalline powder was collected, washed, and used without further purification. Then, a 10 mL solution containing 200 μL of HAc and 70 μL of triethylamine (TEA) (2% HAc, 50 mM TEA) was prepared, and 26 mg (0.0167 mmol) of the acetate zirconium oxo cluster ([Zr_6_O_4_(OH)_4_(Ac)_12_]_2_·(HAc)_6_) was dissolved. In a separate vial, the ligand benzene dicarboxylic acid (H_2_BDC-X, 0.10 mmol) and 28 μL of TEA (0.20 mmol) were dissolved in 10 mL of water. The ligand and metallic aqueous solutions were then mixed at room temperature under stirring. The resulting product was collected by centrifugation and washed three times with water.

#### 2.3.2 Synthesis of MOF-801­(Zr)

An aqueous solution of ZrOCl_2_·8H_2_O (0.75 mmol, 50 mM) was combined with an aqueous solution of sodium fumarate (3 eq., 1 M) at 45 °C. The mixture was stirred at this temperature for 1 h, centrifuged, and the obtained precipitate washed thoroughly with water and EtOH.^30^

#### 2.3.3 Synthesis of MIL-100­(Fe)

MIL-100­(Fe) preparation followed the protocol reported by Guesh et al.,^31^ with some modifications. Briefly, the MOF was synthesized by adding dropwise an aqueous solution of trimesic acid (H_3_BTC) (7.6 mmol, 320 mM) in NaOH (22.8 mmol, 1 M, pH ≈ 11) to an aqueous solution of FeCl_2_·4H_2_O (11.4 mmol, 117 mM, pH ≈ 2.7) under stirring. The final mixture, with a molar ratio of 1.5 Fe/1.0 BTC/3.0 NaOH/880 H_2_O and pH ≈ 5.2, was stirred at room temperature for 24 h. The solid product was recovered by centrifugation, washed with water and EtOH, and dried at room temperature.

#### 2.3.4 Synthesis of MIL-53­(Al)

The synthesis of MIL-53­(Al) was performed by following the protocol reported by Guan et al.^32^ with some modifications. Briefly, 2.9 mmol of terephthalic acid (H_2_BDC) and 1.4 mmol of NaOH were weighed and dissolved in 25 mL of water. Subsequently, 2.9 mmol of AlCl_3_·6H_2_O was dissolved in 10 mL of water. The prepared AlCl_3_ solution was added dropwise to the BDC^2–^ solution under magnetic stirring, and the mixture was further stirred for 24 h at room temperature. A faint yellow material was obtained after centrifugation, washed with water and EtOH, and then dried at 100 °C for 24 h to remove residual moisture.

The corresponding chemical formulas of the tested MOFs, together with their main textural properties, are summarized in Table S2.

### 2.4 Dispersive Extraction Studies with MOFs

To conduct these studies, 10 mg of various MOFs, including UiO-66­(Zr), MOF-801­(Zr), MIL-100­(Fe), and MIL-53­(Al), were placed in 15 mL Falcon tubes. Then, 5 mL of an aqueous solution of herbicides containing 2.5 μg mL^–1^ of each PH (clopyralid, picloram, fluroxypyr, and triclopyr) was added to each tube and stirred at 60 rpm for 30 min. This concentration was selected as the working level for this preliminary screening study and the subsequent optimization extraction experiments with the 3D-printed devices. Afterward, the tubes were centrifugated at 5000 rpm for 5 min. An aliquot of the supernatant (500 μL) was then taken and injected into an HPLC-diode-array detector (DAD) system to assess the retention yield of each MOF.

### 2.5 Design and Fabrication of the 3D-Printed Supports Using FDM

The 3D-printed device was designed using FreeCAD 0.20 software (www.freecadweb.org) and exported as a stereolithography (.stl) file. The .stl file format contains the desired dimensions of the 3D part needed for the 3D printer slicer software (Ultimaker Cura Version 5.4.0, Singapore) to slice the objects and convert them into a G-code file format. The printing parameters were as follows: layer height, 0.24 mm; wall thickness, 1.2 mm; in-fill density, 20% (to avoid void formation); in-fill pattern, grid; printing temperature, 215 °C; build-plate temperature, 60 °C; printing speed, 30 mm/s; fan speed, 100%; print-core nozzle diameter, 0.6 mm; and total printing time, 40 min/device. The device was printed using a 3D printer (Ultimaker S5, Ultimaker, Germany) using FDM technology. Figure 1 presents a schematic representation of the 3D-printed platform. The scaffold comprises a matrix (20 × 14.4 × 14.4 mm^3^) of interconnected parallelepiped cells of two types: long and short. The long cells measure 5.6 × 1.6 × 14.4 mm^3^, while the short cells have dimensions of 1.6 × 1.6 × 14.4 mm^3^. Due to the interconnected structure of these two types of cells, a third type of cell emerges from the top with dimensions of 1.6 × 1.6 × 20 mm^3^. The matrix is attached to a base (16.3 mm × 16.3 mm × 12 mm^3^) featuring a hollow cylinder with a diameter of 5.6 mm, designed to accommodate a magnet that facilitates stirring-assisted sorptive extraction.

**Figure 1:**
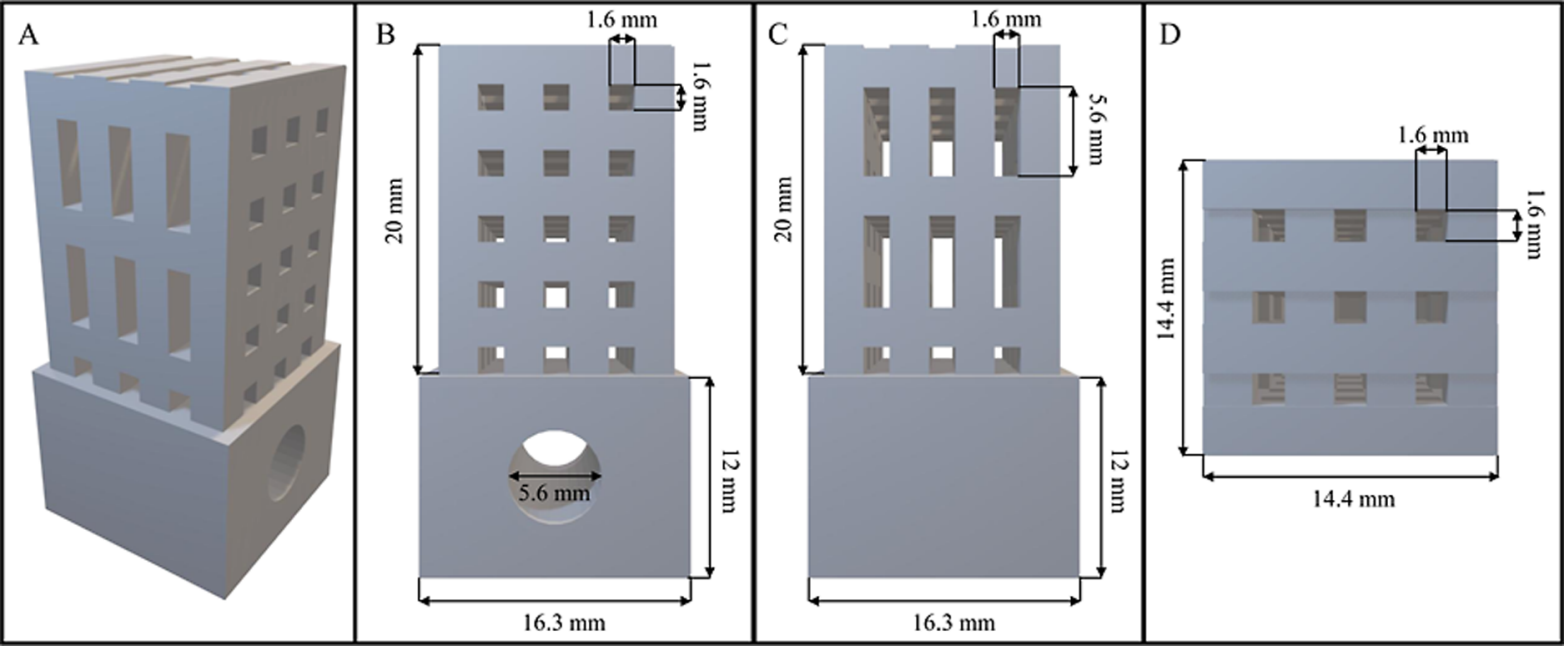
Illustration of 3D-printed device (A) and front (B), lateral (C), and top (D) views. The dimensions of the different parts of the structure are included in the different views.

### 2.6 Modification of 3D-Printed Supports and Immobilization of UiO-66 Analogues in 3D-Printed Devices

Prior to the incorporation of UiO-66 and its derivatives, UiO-66-X (X = NH_2_ and OH), the esterification reaction of 3D-printed parts (see Scheme 1), composed of PLA and wood pine residues, was accomplished according to the procedure described in our previous works.^33,34^ Briefly, the 3D-printed parts were placed in a 50 mL Falcon tube containing 16 g of MA and 8 g of finely ground urea. The tube was shaken and placed in a thermostatic bath at 80 °C for 20 min. After cooling, the derivatized 3D-printed part was removed from the tube, washed with a MeOH/water (2:1, v/v) mixture containing 0.1 M NH_3_, and dried at room temperature. Afterward, to achieve the overgrowth of UiO-66 analogues onto the derivatized 3D-printed parts, the corresponding MOF precursor solutions were added to the preactivated 3D-printed substrate (see Scheme 1). For this purpose, the protocol described for UiO-66 (see above) was properly adopted and extended to its analogues. Briefly, the modified 3D-printed device was immersed in the zirconium cluster precursor solution in water (12 mL of 20 mM in Zr). After 10 min, the ligand solution in water (12 mL of X-BDC, 20 mM) was added dropwise under gentle stirring. The mixture was then stirred for 1 h. The resulting UiO-66-X@devices were rinsed with water three times to remove any unreacted MOF precursors, dried at room temperature for 24 h, and stored in a desiccator until use.

**Scheme 1:**
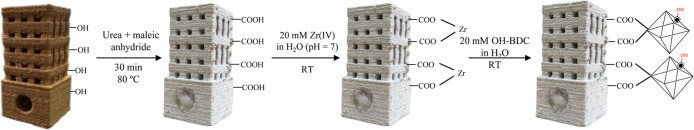
Schematic Illustration of the UiO-66-X@3D-Printed Part Synthesis Step by Step

### 2.7 Extraction Procedure

The extraction of herbicides (clopyralid, picloram, fluroxypyr, and triclopyr) using the 3D-printed device decorated with the selected MOF (UiO-66-OH) was carried out as follows. The 3D-printed device was directly immersed in 25 mL of an aqueous solution (at a defined concentration) or sample, adjusted to pH 3, containing 30% (w/v) NaCl, and then stirred at 400 rpm for 90 min. The nonspecific retained interferences were removed by a washing step with 25 mL of Milli-Q water. Afterward, the 3D-printed device was submerged in 25 mL of MeOH/H_2_O (90:10, v/v) with 100 mM trifluoroacetic acid (TFA) during 30 min. Then, the eluate was evaporated and reconstituted in 1 mL of MeOH/H_2_O (10:90, v/v), and an aliquot was taken, filtered, and injected into the chromatographic system. The HPLC-MS experimental conditions are detailed in the Supporting Information, including mobile phase composition (Table S3) and detection parameters (Table S4).

## 3 Results and Discussion

### 3.1 Stability of the Printed Material under Analytical Conditions and Selection of MOFs

Prior to incorporation of MOFs into the 3D-printed device, it is crucial to assess the stability of the printed material (based on PLA filaments doped with pine residues) in various solvents. For this purpose, the mass and volume changes of the 3D-printed structures were measured after 24 h of immersion in different solvents (Figure S1). The printed material exhibits good stability in most solvents, with variations of less than ±2% after 24 h. However, solvents such as ACN, DMF, and 1 M NaOH significantly compromised the structural integrity of the material, resulting in complete disintegration. In contrast, exposure to DMSO led to increases in mass and volume. Given these results, green synthesis routes for MOFs that use water- or alcohol-based solvents such as MeOH or EtOH were selected. Additionally, the chosen MOFs must also exhibit stability in aqueous environments, as this property is essential for real-world applications in which the samples to be treated are primarily aqueous. Based on these criteria, the following MOFs were selected for testing: UiO-66­(Zr), MOF-801­(Zr), MIL-100­(Fe), and MIL-53­(Al). These MOF candidates were synthesized using green synthetic routes (see Experimental Section) and the resulting powder characterized (see Figure S2). To select the best MOF candidate as a sorbent for PH extraction, a screening study was conducted. For this purpose, retention experiments for these compounds were performed using 10 mg of MOF in 5 mL of ultrapure water solution containing 2.5 μg·mL^–1^ of the target analytes (clopyralid, picloram, fluroxypyr, and triclopyr). For each experiment, the MOF and analyte solution were stirred for 30 min (at 60 rpm) to ensure high material–analyte contact. The quantification of the retained herbicides was obtained by injecting the filtered supernatant into an HPLC–DAD system (see Experimental Section in Supporting Information). Figure 2 presents the results in terms of retention efficiency for each analyte with the different MOFs tested. Dispersive extraction studies revealed significantly larger retention efficiencies for the more hydrophobic analytes, fluroxypyr and triclopyr, over the more hydrophilic clopyralid and picloram. In addition, UiO-66­(Zr) and MIL-100­(Fe) presented higher retention efficiencies for these hydrophobic analytes compared to MOF-801­(Zr) and MIL-53­(Al). This improved retention can be attributed to the larger accessible porosity of the UiO-66­(Zr) and MIL-100­(Fe) materials, considering the molecular sizes of the target herbicides (width < 6.7 Å, length < 8.9 Å, Figure S3). The lower retention observed in MIL-53­(Al) may be related to its characteristic flexibility, which can prevent analyte extraction upon closed phase transformation during experimental conditions. Among UiO-66­(Zr) and MIL-100­(Fe) candidates, UiO-66­(Zr) exhibited a superior retention of the more challenging polar herbicides. Additionally, UiO-66 is particularly convenient since its plausible multivariate composition can be exploited as a modular chemical platform to prepare polar and hydrophilic derivatives such as UiO-66-NH_2_ and UiO-66-OH, which will also be investigated. These UiO-66 derivatives feature organic linkers different than H-BDC while maintaining the isoreticular network structure (see Figure S4). Therefore, the UiO-66 topology was chosen as the most suitable sorbent for further incorporation into the 3D-printed supports.

**Figure 2:**
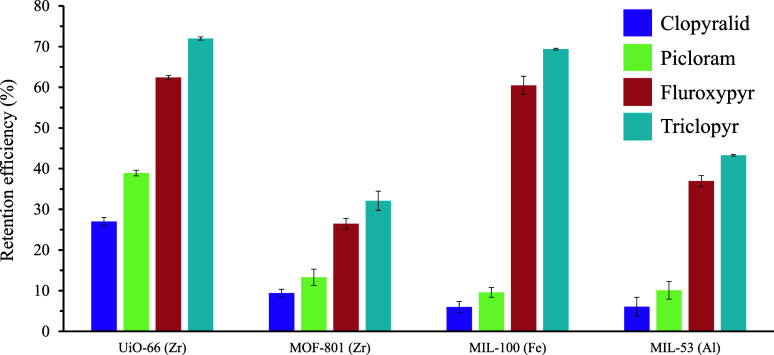
Retention efficiency of PHs using dispersive extraction studies using several MOFs.

### 3.2 In Situ Growth of UiO-66­(Zr) onto 3D-Printed Structures

The geometry of the 3D-printed device was designed to provide an open and mechanically stable structure that maximizes contact between the MOF-coated surface and the aqueous solution. The interconnected lattice enhances surface area and mass transfer under stirring-assisted conditions while maintaining sufficient structural integrity for repeated use. To achieve the growth of UiO-66­(Zr) onto the 3D-printed scaffold, the PLA–wood substrate was first treated by an esterification process with MA to generate carboxylate groups (as described in Experimental Section). FT-IR spectroscopy and XRPD studies were employed to confirm successful pretreatment (see Figures S5 and S6). Overall, the PLA–wood filament FT-IR spectrum is similar to that of neat PLA (Figure S5). The characteristic peak at ca. 1745 cm^–1^, corresponding to the stretching vibration of C=O, along with peaks at 1090 and 1180 cm^–1^ attributed to the stretching vibrations of C–O, is clearly distinguishable. An absorption band between 2850 and 3000 cm^–1^, due to the asymmetric and symmetric stretching vibrations of –CH, is also observed. Moreover, a band around 3300 cm^–1^ was identified, which was attributed to the lignocellulose material components. After MA treatment, the peak at 1745 cm^–1^ shows an increase and noticeable broadening, attributed to the superposition of the C=O ester peaks from MA and PLA. Furthermore, the appearance of a new peak at 1625 cm^–1^, corresponding to the cyclic C=C stretching of anhydride, confirms the esterification reaction between the PLA–wood substrate and MA.^35,36^

In addition, XRPD patterns of neat PLA, PLA–wood, and treated PLA–wood composite 3D-printed substrates were also measured (Figure S6). As observed, the PLA–wood composite exhibits a broad diffraction peak centered at 2θ = 16°, characteristic of amorphous PLA, with a shoulder at 22.4° attributed to the cellulose peak. After MA treatment, the substrate exhibits well-defined peaks at 16.9° and 19.4°, indicating the formation of a crystalline structure in PLA, the primary component of the composite, which can be attributed to enhanced polymer chain packing within the crystal lattice.^37,38^ Additionally, small peaks associated with cellulose are observed at 22.6°.

Subsequently, UiO-66­(Zr) growth onto the treated 3D-printed part was carried out via direct exposure of the support to the MOF precursor solutions. In this context, the preparation protocol for UiO-66 MOFs in powder form (see Experimental Section) was adapted by varying the volume of the metal/ligand MOF precursor solutions (5, 12, and 15 mL each). To monitor the formation of the UiO-66@3D-printed device, the XRPD pattern of the 3D-printed surface-removed crystals was compared to that of UiO-66­(Zr) crystalline powder obtained from a bulk synthesis (see Figure S7). As observed, 12 mL provided a more crystalline MOF coating while avoiding unnecessary excess of reagents and was therefore selected for further experiments. The same protocol was applied to prepare UiO-66-NH_2_ and UiO-66-OH, yielding XRPD patterns identical to those of the parent material, thus confirming that these MOFs are isoreticular to UiO-66­(Zr). In addition, the porosity of the synthesized UiO-66-type materials was verified by N_2_ sorption measurements revealing a fully accessible porosity characteristic of the UiO-66 structure (see Figure S8).

To further confirm the successful incorporation of MOF onto the 3D-printed device, SEM micrographs were taken from a top view of the structure (located in one of the lateral sections of the device) of an uncoated 3D-printed device (A–C) and coated with UiO-66-OH (as a representative example) (D–F) at different magnifications (Figure 3). The SEM images (Figure 3A,B) of the surface of the uncoated 3D-printed PLA–wood material show areas of the PLA matrix with internal voids, likely caused by the vaporized moisture in the wood or trapped air remaining in the medium during the mixing of wood fibers with PLA.^39^ Also, a few wood fibers from the PLA matrix were observed, probably attributed to the insufficient interfacial strength between the fibers and the matrix (Figure 3C).^39,40^ On the other hand, the SEM images of the MOF-coated 3D-printed device confirm uniform coverage of the device’s surface (Figure 3D,E). The high-magnification SEM image (Figure 3F) shows that MOF particles, composed of spheroidal-shaped aggregates, are located on the surface of the polymer matrix. To gain insight into the thickness of the MOF coating, a 1 cm × 1 cm square rod was 3D-printed and subsequently coated. A SEM image of the cross section of the coated 3D-printed part shows that the coating thickness was approximately 58 μm (Figure 3G).

**Figure 3:**
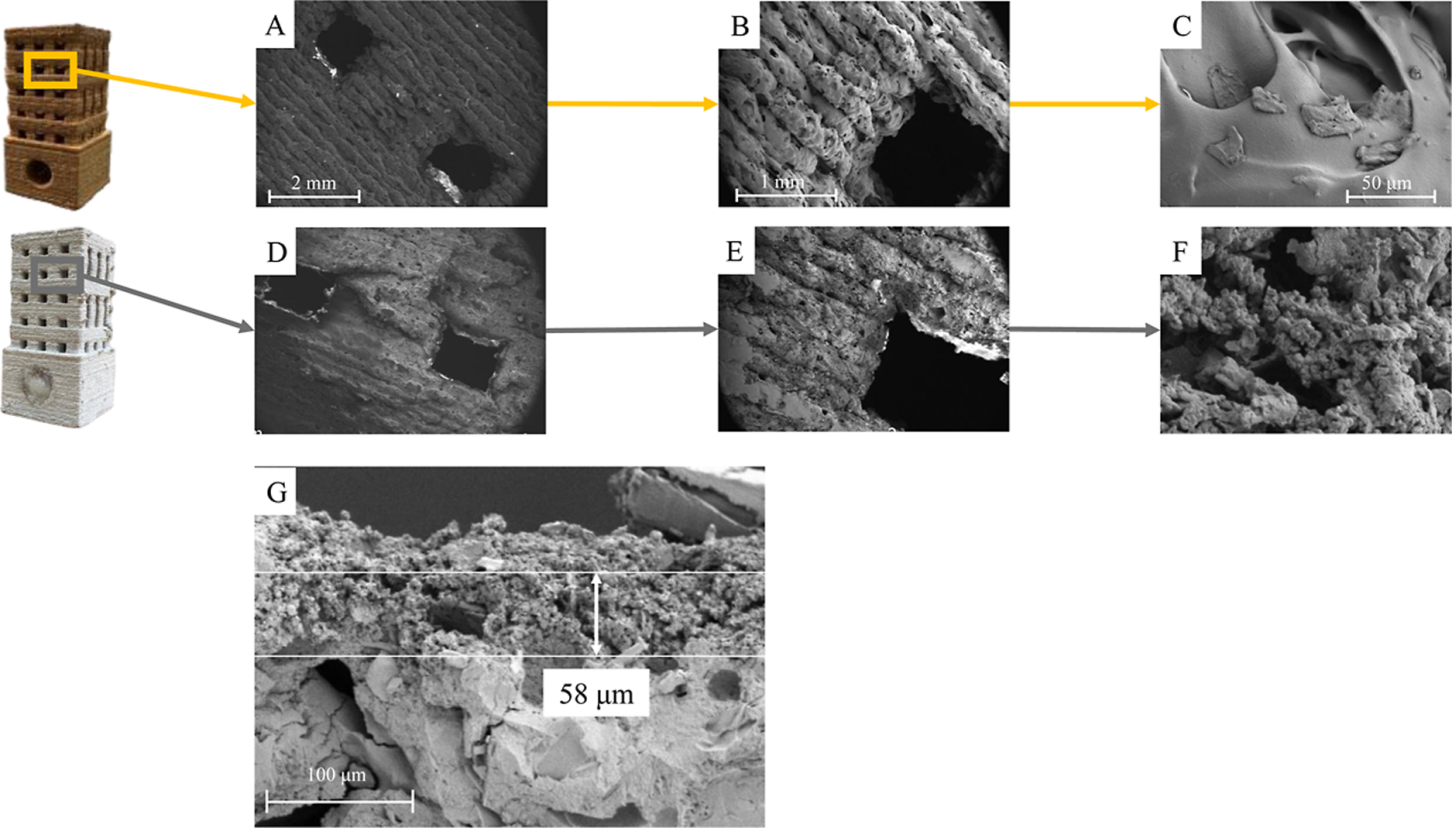
SEM images of the top view of an uncoated 3D-printed device (A–C) and 3D-printed device coated (D–F) with UiO-66-OH (as a representative example) at different magnifications. SEM image of the cross-sectional view of a 3D-printed part (1 cm × 1 cm square rod) coated with UiO-66-OH (G).

Energy-dispersive X-ray (EDS) analysis was performed to unequivocally confirm the incorporation of the MOF onto the 3D-printed scaffold. As depicted in Figure S9, the EDS spectrum shows the presence of Zr, consistent with the MOF structure.

The XRPD patterns of the 3D-printed PLA–wood material carboxylated (esterified) and coated with MOF were also obtained. As shown in Figure 4, the most intense diffraction peaks present in the UiO-66-OH diffractogram are clearly visible in the XRPD pattern of the coated device. Moreover, these diffraction peaks were also detected in XRPD measurements taken at various locations of the UiO-66-coated 3D-printed device, confirming the homogeneous distribution of UiO-66-OH crystals on its surface. Similar findings were observed for the other UiO-66-type frameworks studied.

**Figure 4:**
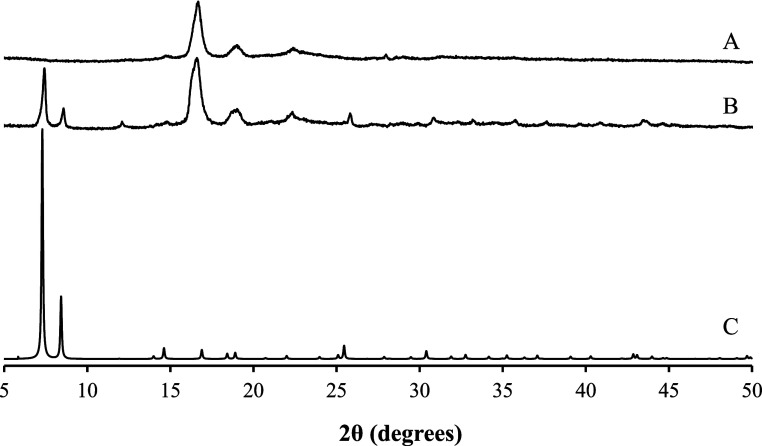
XRPD patterns of the 3D-printed device before (A) and after coating with the MOF (B), compared to the simulated pattern of UiO-66-OH (C).

The growth of UiO-66 onto the 3D-printed device was further confirmed by FT-IR analysis. As shown in Figure S10, the FT-IR spectrum of the 3D-printed device coated with UiO-66-OH shows the characteristic absorption bands of the pristine MOF, suggesting its successful incorporation onto the 3D-printed support. In particular, the bands observed around 1350–1600 cm^–1^ correspond to the asymmetric and symmetric stretching vibrations of C=C and coordinated carboxylate C–O groups of the OH-BDC linker; the band located at 1240 cm^–1^ is attributed to the C–O stretching vibration of phenolic hydroxyl groups, while the bands observed around 750–700 cm^–1^ can be associated with the out-of-plane aromatic C–H vibrations, and the lower-frequency region (650–500 cm^–1^) is mainly related to Zr–O vibrations.^41–43^ Additionally, a broad absorption in the 3200–3500 cm^–1^ region is assigned to OH stretching vibrations, consistent with the presence of a hydroxyl-functionalized linker. After confirming the incorporation of UiO-66’s onto 3D-printed devices, the resulting scaffolds were tested for their ability to retain the target herbicides. In all experiments, an aqueous solution containing the four analytes (25 mL, 2.5 μg mL^–1^) was used under stirring at 200 rpm for 60 min. The retention efficiencies from this study are listed in Table 1. A clear trend was observed corresponding to higher retention values for the 3D-printed devices functionalized with UiO-66-NH_2_ and UiO-66-OH, compared with the parent UiO-66. UiO-66-OH demonstrated the highest retention efficiency across all tested analytes, ranging from 33% to 79%. This improved efficiency compared to UiO-66-NH_2_ may be related to the more versatile −OH group, which can act as both a hydrogen-bond donor (via H) and a hydrogen-bond acceptor (via O). This dual capability allows UiO-66-OH to interact strongly with the polar functional groups in the target herbicides. In addition, the slightly bulkier nature of −NH_2_, together with its lower electronegativity, may hinder access and diffusion to the pores. Overall, these results indicate that the hydrophilic nature of the ligand plays a key role in improving the retention of polar analytes (such as clopyralid and picloram). Although this interpretation is tentative, it is consistent with previous studies reporting improved adsorption and extraction performance of hydroxyl-functionalized UiO-66 materials for both relatively polar organic compounds and ionic species, such as neonicotinoid insecticides,^44^ Th­(IV),^45^ and As­(III).^46^ Consequently, UiO-66-OH and, by extension, the 3D-printed parts coated with it were selected for further studies. For comparison purposes, retention values obtained using the untreated PLA–wood support and the PLA–wood support treated with MA prior to MOF growth were also included. Both supports showed low retention values (<17%), confirming that the extraction performance mainly arises from the MOF layer, while the 3D-printed structure acts primarily as a mechanical support for MOF growth.

**Table 1:** Influence of Organic Ligands of the Synthesized UiO-66-Based MOFs on the Retention Values of Herbicides

	retention (%) ± SD (*n* = 3)				
analyte (PHs)	PLA–wood	PLA–wood–MA	UiO-66	UiO-66-NH_2_	UiO-66-OH
clopyralid	8.1 ± 0.7	6.1 ± 0.6	27 ± 1.4	31.4 ± 0.6	33.0 ± 1.3
picloram	12.2 ± 0.6	9.2 ± 0.5	38.9 ± 0.7	44.1 ± 1.2	48.0 ± 1.4
fluroxypir	15.3 ± 0.8	13.4 ± 0.7	62.4 ± 0.5	65.9 ± 0.7	68.5 ± 0.9
triclopyr	16.4 ± 0.7	15.2 ± 0.6	72.0 ± 0.4	74.8 ± 0.3	79.0 ± 1.4

Prior to evaluation of the extraction parameters with the selected UiO-66-OH, the overgrowth reproducibility was assessed. For this purpose, 6 devices were fabricated and processed as outlined in Scheme 1. The Zr content was determined in all the samples by ICP–MS by digestion of the MOF@3D-printed support, yielding 0.0520 ± 0.0008 wt % (which corresponds to ca. 0.38 wt % MOF). The device-to-device variation showed a relative standard deviation (RSD) value of 1.5%, providing satisfactory reproducibility. Based on this MOF weight fraction and the mass of each 3D-printed support (ca. 5 g), the MOF loading was estimated to be about 19 mg of MOF per device.

### 3.3 Optimization of Extraction Parameters

To examine the analytical performance of the 3D-printed device decorated with UiO-66-OH as the extraction sorbent, several parameters that can influence the extraction efficiency were investigated. For these optimization studies, an aqueous solution containing 2.5 μg·mL^–1^ of each PH was used as a test mixture (Figures S11 and S12), and the experiments were monitored using the HPLD–DAD system. Optimal results were obtained under the following experimental conditions: (i) loading solution at pH 3 with 30% (w/v) NaCl; (ii) retention time, 90 min; (iii) stirring rate, 400 rpm; (iv) elution solvent, MeOH/H_2_O (90:10, v/v) with 100 mM trifluoroacetic acid (TFA); and (v) elution time, 30 min, respectively.

Next, the possibility of concentrating the final extract through evaporation and redissolution was evaluated. For this purpose, methanolic water solutions containing the target analytes were evaporated at room temperature under a nitrogen stream and then reconstituted in smaller volumes of the mobile phase. Using a final reconstitution volume of 1 mL, no significant analyte losses were observed, with recoveries ranging from 79% to 95%.

The reusability of the adsorbent is a key aspect from both environmental and economic perspectives. Therefore, this parameter was evaluated in the 3D-printed device coated with UiO-66-OH by repeatedly applying the optimal extraction protocol to an aqueous standard solution of target analytes at 500 μg L^–1^ (Figure S13). The obtained results indicate a stable recovery over the first eight reuses. In addition, the structural stability of the MOF-coated device was assessed by XRPD after the reuse cycles (Figure S14). The corresponding diffraction patterns retained the main characteristic reflections of the fresh material, confirming that the crystalline framework of the UiO-66-based MOF remains largely preserved after repeated use.

### 3.4 Analytical Features

The analytical performance of the optimal extraction protocol using the UiO-66-OH-coated 3D-printed devices in combination with HPLC-MS/MS was established (Table 2). A good linearity was achieved for all analytes, with correlation coefficient (*r*) > 0.999 across the concentration range (10–500 μg L^–1^). The instrumental limits of detection (LOD) and quantification (LOQ) were established at signal-to-noise (S/N) ratios of 3 and 10, respectively, giving values ranging from 0.04 to 0.08 μg L^–1^ and from 0.13 to 0.26 μg L^–1^. The LOD and LOQ values for the proposed method were calculated considering the obtained instrumental values and enrichment factor (25), resulting in ranges of 0.0016 to 0.0032 and 0.005 to 0.011 μg L^–1^, respectively. The preparation reproducibility of MOF-coated 3D-printed devices was also investigated with aqueous solution samples containing 50 μg L^–1^ of each herbicide. As shown in Table 3, the RSDs for preparation reproducibility ranged from 0.3 to 3.3% (*n* = 3) in one batch and from 4 to 12% (*n* = 3) among different batches.

**Table 2:** Analytical Figures of Method Using the UiO-66­(OH)-Coated 3D-Printed Device with HPLC-MS/MS for the Analysis of PHs

				precision (RSD, %)	
analyte	instrumental working range (μg·L^–1^)	method LOD (μg·L^–1^)^a^	method LOQ (μg·L^–1^)^a^	intrabatch^b^	interbatch^c^
clopyralid	10–500	0.0024	0.008	3.3	12
picloram	10–500	0.0024	0.008	1.8	10
fluroxypyr	10–500	0.0016	0.005	0.7	4
triclopyr	10–500	0.0032	0.011	0.3	6

a Values obtained applying the optimized extraction protocol.

b Interday values (*n* = 3) using a single MOF-coated 3D-printed device.

c Interbatch values (*n* = 3) using different MOF-coated 3D-printed devices.

**Table 3:** Recoveries of Herbicides in Spiked Water Samples Using the Proposed Method^a^

		recovery (%) ± SD			
sample	spiked level (μg L^–1^)	clopyralid	picloram	fluroxypyr	triclopyr
tap water	50	77 ± 4	81 ± 2	102 ± 3	101 ± 2
	100	78 ± 3	81 ± 4	94 ± 3	102 ± 6
water reservoir	50	76 ± 4	82 ± 2	95 ± 2	98 ± 3
	100	78 ± 5	83 ± 3	94 ± 4	96 ± 6
seawater	50	78 ± 4	79 ± 1	98 ± 1	104 ± 2
	100	76 ± 7	82 ± 2	96 ± 1	107 ± 3
wastewater	50	73 ± 4	75 ± 2	94 ± 3	95 ± 2
	100	74 ± 3	76 ± 3	97 ± 4	97 ± 8

a Recovery (%) ± SD (*n* = 3).

Furthermore, the proposed method was compared with previously reported methodologies for the extraction and determination of PHs in water samples (Table S5). Most procedures rely on SPE with commercial sorbents, generally providing recovery values comparable to those obtained in this work, although significantly lower recoveries (ca. 42%) have been reported in one study. The developed method exhibited lower or comparable LODs compared to most reported methods. Additionally, the developed extraction device showed suitable reusability (up to 8 cycles), in contrast to many reported methods in which this parameter is either not evaluated or limited by the single-use nature of commercial SPE cartridges. Moreover, the preparation of the extraction device is cost-effective, with an estimated cost of approximately 0.3€ per printed part, increasing to about 0.5€ per device after MOF incorporation. This is considerably lower than the cost associated with commercial SPE cartridges. Overall, the proposed method combines excellent analytical performance with good reusability and low cost.

### 3.5 Application to Real Samples

To assess the applicability of the proposed analytical method, extraction and determination of target compounds in environmental water samples were performed. No target herbicides were detected in any of the samples studied. Thus, in order to establish the accuracy of the method, the samples were spiked with the four herbicides at two concentration levels (50 and 100 μg·L^–1^). The results obtained are summarized in Table 3. The recoveries for herbicides in spiked water samples were 73–107%, which confirm the effectiveness of the proposed method for extracting and analyzing these compounds in water matrices. Figure S15 presents multiple reaction monitoring (MRM) chromatograms of a tap water sample (as representative sample) spiked with target herbicides, following the proposed extraction procedure.

## 4 Conclusions

This paper demonstrates the feasibility of using sustainable 3D-printed supports for the incorporation of MOFs synthesized through green routes, enabling the development of sorbents for the extraction of pollutants from water samples and providing a more environmentally friendly alternative to conventional approaches. A screening study of various MOF materials obtained under green conditions was conducted in dispersive mode to evaluate their sorption performance. Among them, the UiO-66’s family emerged as the most suitable candidates for capturing the target analytes. UiO-66-type MOFs were successfully incorporated into 3D-printed scaffolds fabricated by FDM via simple esterification (carboxylation) process applied to PLA–wood filaments. The in situ UiO-66’s growth procedure onto the printed parts was successfully achieved at room temperature and in the absence of organic solvents, conditions typically required in conventional MOF synthesis. Characterization of the resulting UiO-66-X@3D-printed devices confirmed successful MOF integration. Among them, UiO-66-OH@3D-printed devices showed the highest retention efficiency and achieved satisfactory recoveries (73% to 107%) when applied to the extraction of PHs in environmental water samples. The devices demonstrated good reproducibility (RSD < 12%), reusability (up to eight uses), and a highly cost-effective sorbent production, with the estimated costs of around 0.3€ per print and <0.50€ per device incorporating the MOF. Finally, we demonstrate a simple and affordable strategy for preparing sustainable MOF@3D-printed devices. This strategy can be extended to other MOFs and functional materials, representing a promising approach for the efficient extraction and/or removal of hazardous compounds in environmental water samples and other complex matrices. Although the current fabrication procedure involves manual steps and moderate production times (ca. 40 min), it is compatible with scalable additive manufacturing. This approach provides flexibility for future scaling through parallel or automated production, while further optimization of the functionalization and MOF growth steps could enable batch processing. Moreover, device reusability helps mitigate fabrication time constraints. In any case, the integration of 3D printing with sustainable fabrication and green MOF-based systems opens new opportunities for the development of next-generation sorbents and strategies for environmental analysis and remediation.

## Supplementary Material



## Notes

Experimental section; structures and physicochemical properties of PHs; characterization of the tested MOFs; UHPLC-MS/MS mobile phase composition and detection parameters for target herbicides; results and discussion, including stability of the 3D-printed material in organic solvents; XRPD patterns of the synthesized MOFs; structural size comparison; XRPD and FTIR characterization of MOFs and 3D-printed scaffolds; nitrogen sorption isotherms; EDS and FTIR analysis of coated devices; optimization of extraction parameters; retention efficiency and recovery studies; reusability assessment by recoveries and XRPD; comparison with reported methods; MRM chromatograms of spiked tap water samples; and references (PDF)
