# Supplementary material for: Sustainable 3D-Printed Supports Coated with Zirconium-Based Metal–Organic Frameworks for Picolinic Herbicide Extraction
Source: ACS Sustain Chem Eng. 2026 Apr 22;14(17):8436–45. doi: 10.1021/acssuschemeng.6c01944 (PMC13148721; doi:10.1021/acssuschemeng.6c01944)
Supplement: Supplementary file 1 [file sc6c01944_si_001.pdf]

SUPPLEMENTARY INFORMATION FOR

Sustainable 3D-printed supports coated with zirconium-based metal-organic frameworks for picolinic herbicides extraction

Alejandro Gil-Aparicio[a], Jana Maren-Glatz[b], Jesús Cases-Díaz[b] Enrique Javier Carrasco-Correa[a], Mónica Giménez-Marqués[b], José Manuel Herrero-Martínez[a]*

[a]*Department of Analytical Chemistry, University of Valencia,* Av. Vicent Andrés Estellés, 19*, 46100-Burjassot, Valencia, Spain.*

[b]*Instituto de Ciencia Molecular, University of Valencia, C/ Catedrático José Beltrán Martínez, 2, 46980-Paterna, Valencia, Spain.*

*Corresponding author: Dr. José Manuel Herrero-Martínez

Tel.: +34963544062

Fax: +34963544436

e-mail: jmherrer@uv.es

Dr. Mónica Giménez-Marqués

Tel.: +34963544418

e-mail: monica.gimenez-marques@uv.es

Table of contents

Page S4-S6. Experimental section

Page S7. Structures of PHs and physicochemical properties (Table S1)

Page S8. Characterization features of tested MOFs (Table S2)

Page S9. UHPLC-MS/MS mobile phase composition and detection parameters for the target herbicides (Tables S3 and S4)

Page S10-S21. Results and discussion

Page S10. Stability of 3D-printed material in different organic solvents (Figure S1) and experimental and theoretical XRPD pattern of the synthesized powder MOFs (Figure S2)

Page S11. Structural sizes of PHs and UiO-66 cavities (Figure S3)

Page S12. XRPD patterns of UiO-66 typology MOFs (Figure S4), FTIR spectra and XRPD patterns of 3D-printed scaffolds made from neat PLA, PLA-wood composite filaments and treated with MA (Figure S5 and S6, respectively)

Page S13. XRPD patterns of UiO-66 crystals removed from 3D-printed surface (Figure S7) and nitrogen sorption isotherm for UiO-66-OH material (Figure S8)

Page S14. EDS analysis of UiO-66-OH-coated 3D-printed device (Figure S9) and FTIR spectra of the 3D-printed device before and after coating with UiO-66-OH (Figure S10)

Page S15-S19. Optimization of extraction parameters

Page S17. Influence of several variables on retention efficiency of PHs (Figure S11)

Page S18. Influence of eluent on the PH recoveries (Figure S12) and reusability study of the extractant units evaluated through recoveries (Figure S13)

Page S19. Reusability of the extraction units assessed by XRPD patterns (Figure S14).

Page S20. Comparison of MOF@3D-printed device–HPLC-MS with reported methods for PH analysis (Table S5)

Page S21. MRM chromatograms of tap water spiked with PHs after applying the proposed extraction procedure (Figure S15)

Page S22-23. References

EXPERIMENTAL SECTION

Materials and instrumentation. Commercial PLA Pine Smartil filaments were supplied by Smart Materials, S.A. (Jaén, Spain). This material is a commercial PLA–wood composite, for which the exact quantitative composition is not disclosed by the manufacturer. This filament (brown colour, 2.85 mm i.d.) was characterized by the producer in terms of printing conditions (printing temperature: 200-230 °C and bed temperature: 40–60 °C). It is also described as biodegradable and compostable. To modify the 3D-printed parts, the following reagents and solvents were used: maleic anhydride from Thermo Scientific (Vilnius, Lithuania), urea from Labkem (Barcelona, Spain), and methanol (MeOH) acquired from VWR. Metal salts such as aluminium chloride hexahydrate, zirconium tetrachloride, iron (III) chloride hexahydrate and the organic ligands (terephthalic acid (BDC), 2-aminoterephthalic acid (NH_2-BDC), 2-hydroxyterephthalic acid (OH-BDC) and trimesic acid (BTC) were obtained from Merck-Sigma-Aldrich (Darmstadt, Germany). Clopyralid, picloram, fluroxypyr and triclopyr were also purchased from Merck-Sigma-Aldrich. Table S1 shows the complete names, structures and the main physicochemical properties of the studied herbicides. Stock solutions of each analyte were performed at 1000 $mg \cdot L^{-1}$ in MeOH. Then, a mixture of all of them (100 $mg \cdot L^{-1}$) was done by proper dilution from the stock solution in Milli-Q water. All the solutions were stored at 4 °C until use. All organic solvents (e.g. MeOH and acetonitrile (ACN)) as well as other reagents (NaOH, ammonia, formic and trifluoroacetic acid (TFA)) were of analytical grade, unless otherwise stated, obtained from Panreac/AppliChem (Barcelona, Spain). Deionized water was obtained from the Milli-Q purification system (Millipore Ibérica, Madrid, Spain) with a resistivity lower than 18 MΩ cm^{-1}.

A Ultimaker S5 3D-printer (Ultimaker B.V., Geldermalsen, The Netherlands) was used for the fabrication of 3D-printed parts. A rotator stirrer with carousel type design LBX RD80

series from IKA was used to conduct the extraction studies in dispersive mode. For material characterization, scanning electron microscopy (SEM) micrographs of 3D-printed materials were taken with a field emission scanning electron microscope with focused ion beam (FIB-SEM) model SCIOS 2 (ThermoFisher Scientific), which was coupled to an energy dispersive spectrometer (EDAX, Oxford Ultim Max 170) in order to provide chemical analysis. Powder X-ray Diffraction (p-XRD) spectra of powdered MOFs and 3D-printed materials were acquired in a D8 Advance A25 X-ray diffractometer (Bruker, Berlin, Germany). Attenuated total reflection (ATR) Fourier-transform infrared (FTIR) spectra of materials were recorded using a Cary 630 FTIR spectrometer equipped with a diamond ATR accessory from Agilent Technologies (Waldbronn, Germany). FTIR spectra acquisitions were performed from 4000 to 400 cm−1 (with 4 cm−1 resolution, and an average of 25 scans). The zirconium content in UiO66-based materials and 3D-printed devices coated with UiO-66-OH was determined using a 7900 inductively coupled plasma mass spectrometer (ICP-MS) (Agilent Technologies, Waldbronn, Germany), after acidic microwave digestion using a mixture nitric acid-water. The determination of the Zr content was repeated several times.

During optimization stage, chromatography separation of PHs was achieved using a liquid chromatograph Agilent 1260 Infinity II HPLC instrument (Agilent Technologies) equipped with a quaternary gradient pump, a solvent degasser system, an autosampler, and a diode-array detector (DAD). The column was a Kinetex EVO C18 100 Å column (4.6 × 150 mm, 5 µm particle size) from Phenomenex (Torrance, California, USA). The chromatographic system was controlled by an OpenLAB CDS LC ChemStation from Agilent (B.04.03). Analytes separation was carried out at 1 $mL{\cdot}min^{-1}$ using a mobile phase consisted of 0.1% formic acid in Milli-Q water (eluent A) and HPLC grade MeOH containing 0.1% formic acid (eluent B). Gradient elution conditions started with 90% A and 10% B, followed by a decrease to 10% A in 5 min, which was hold for an additional 3 min. This was followed by

a return to the initial mobile phase composition within 0.10 minutes. The column was then equilibrated for 1.90 min, therefore the total run time of the analysis was 10 min. The column was kept at room temperature during the separation. The injection volume was 80 µL, and the detection wavelength was set at 220 nm for the fluroxypyr, and 230 nm for the rest of herbicides. All samples were filtered before their injection with 0.45 µm PTFE filters.

For sample analysis, an Acquity UPLC Waters liquid chromatograph equipped with a triple quadrupole mass spectrometry (MS/MS) detector was used. A Waters BEH C18 column was employed (2.1 × 50 mm, 1.7 µm particle size), and separation was performed at 35°C under gradient conditions using a mixture of ACN and water, both containing 2 mM ammonium formate and 0.2% formic acid (see Table S2). Mass spectrometer was operated in negative ion mode and multiple reaction monitoring (MRM) mode. The operating parameters were as follows: the ion source temperature was 400°C and the gas pressures of the curtain gas, ion source gas 1, and ion source gas 2 were 35, 60, and 60 psi, respectively. The specific transition ions monitored for each herbicide are given in Table S3.

Samples. Four real water samples were collected from different sources: tap water, water reservoir, seawater and wastewater. This last sample was directly collected from a sewer located at the University of Valencia (Burjassot, Spain) campus on different days during February 2024. Sampling was performed using a polyethylene flask attached to an aluminum telescopic pole. The collected samples were transferred to pre-cleaned glass bottles, stored in a refrigerator, and protected from light to prevent potential analyte degradation. The same type of bottles and storage conditions were applied to all other analyzed samples. The samples were pre-filtered using 0.45 µm nylon membrane filters to remove solid particulates. For wastewater, pre-centrifugation was required before filtration. All samples were then stored in the dark at −20°C until analysis.

Table S1. Structures and physicochemical properties of the studied PHs.[1]

Compound	Structure	Log $P_{O/W}$[1]	pK_a[1]
Clopyralid	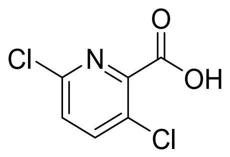	-2.63	2.01
Picloram	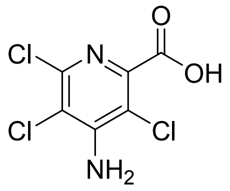	-1.92	1.8
Fluroxypyr	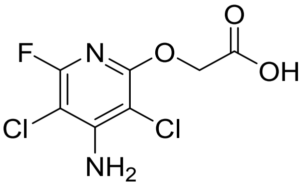	0.04	2.94
Triclopyr	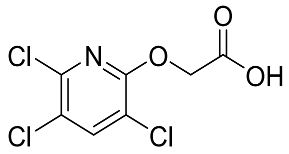	-0.45	3.97

Table S2. Characterization features of tested MOFs.

MOF	MOF chemical formula	BET Surface area ($m^2 \cdot g^{-1}$)	Pore cavity (Å)	References
UiO-66(Zr)*	$Zr_6O_4(OH)_4[O_2C–C_6H_4–CO_2]_6$	930	7–9	[2, 3]
MOF-801(Zr)	$Zr_6O_4(OH)_4[O_2C–CH=CH–CO_2]_6$	755	5-7	[4, 5]
MIL-100(Fe)	$Fe_3O(H_2O)_2(OH)[C_6H_3(CO_2)_3]_2$	1900	10-12	[6]
MIL-53(Al)	$Al(OH)[O_2C–C_6H_4–CO_2]$	980	10-12	[7]

*The chemical formulas of UiO-66 derivatives were as follows: UiO-66-NH_2: $Zr_6O_4(OH)_4[O_2C–C_6H_3(NH_2)–CO_2]_6$; and UiO-66-OH: $Zr_6O_4(OH)_4[O_2C–C_6H_3(OH)–CO_2]_6$.

Table S3. UHPLC-MS/MS mobile phase composition. Components: (A) water (containing ammonium formate 2 mM and 0.2% formic acid) and (B) ACN (containing ammonium formate 2 mM and 0.2% formic acid).

**Time (min)**	**%B**
0	0
0.75	0
1	25
5	80
14	100
15	100
15.1	0
20	0

Table S4. UHPLC-MS/MS detection parameters for the target herbicides.

**Analyte**	**Quantifier transition ion, Q (*m/z*)**	**Qualifier transition ions, q (*m/z*)**
**Clopyralid**	190 → 146	190 → 35
**Picloram**	241 → 197	239 → 195
**Fluroxypyr**	253 → 195	253 → 233
**Triclopyr**	254 → 196	254 → 218

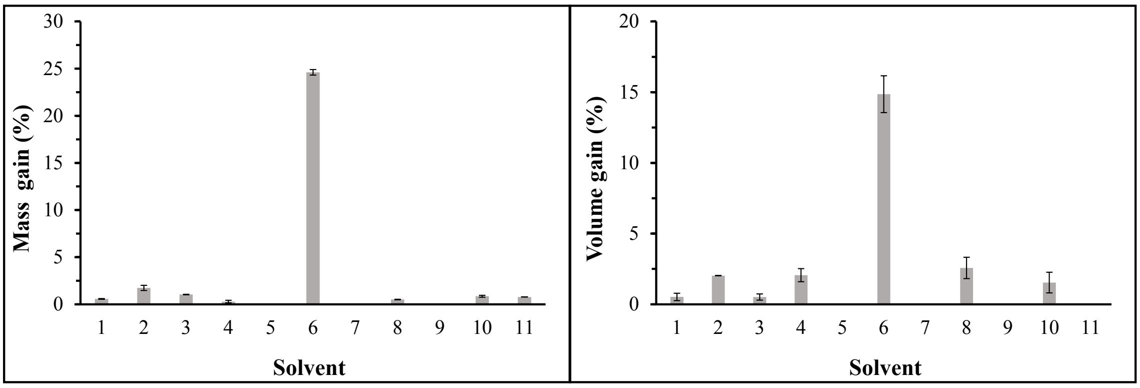

Figure S1. Stability of 3D-printed material in different organic solvents over 24 h at room temperature: (1) H_2O, (2) MeOH, (3) EtOH, (4) IPA, (5) ACN, (6) DMSO, (7) DMF, (8) HCl 1M, (9) NaOH 1 M, (10) NH_3 1 M and (11) HAc 1 M. Error bars represent the standard deviations obtained with three replicate measurements. Where not visible, the error bar is smaller than the size of the data point marker.

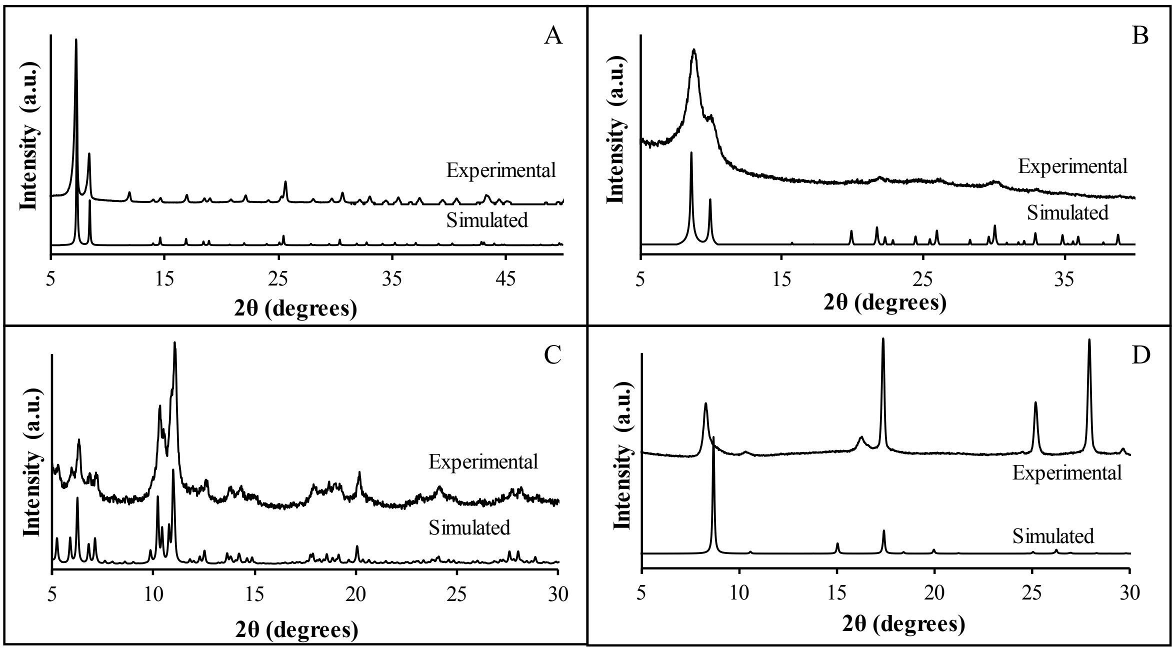

Figure S2. Experimental and simulated XRPD pattern of the synthesized powder MOFs: UiO-66(Zr) (A), MOF-801(Zr) (B), MIL-100(Fe) (C), and MIL-53(Al) (D).

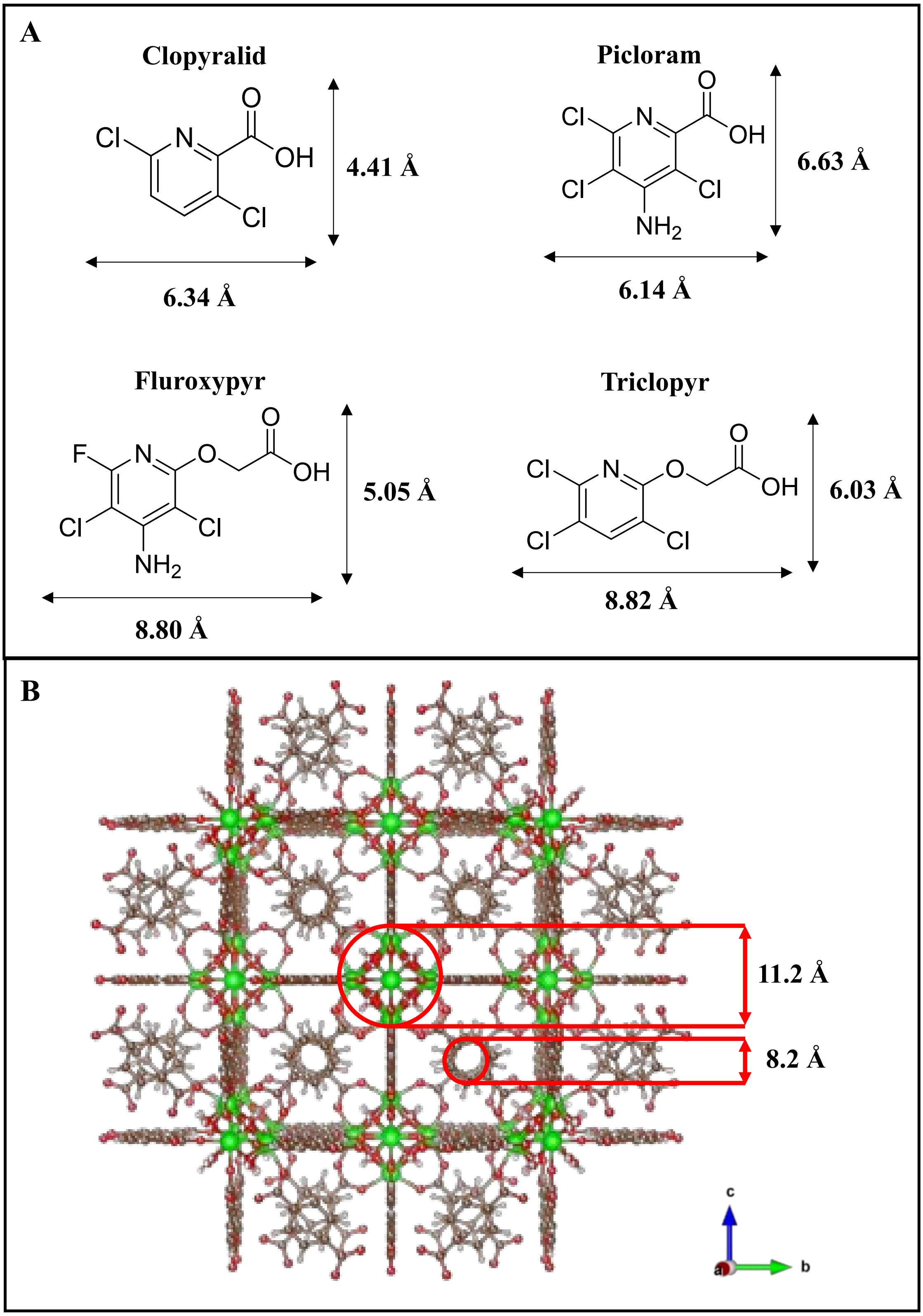

Figure S3. Molecular sizes of the target PHs (A) and UiO-66 (B). The structures of analytes were obtained from Chem3D and ChemDraw. To obtain the sizes, an energetic minimization was done using MM2 model at 300K. (Minimum RMS Gradient = 0.0100). Colors' code: O atoms (red), H atoms (pale pink), C atoms (brown) and Zr atoms (green).

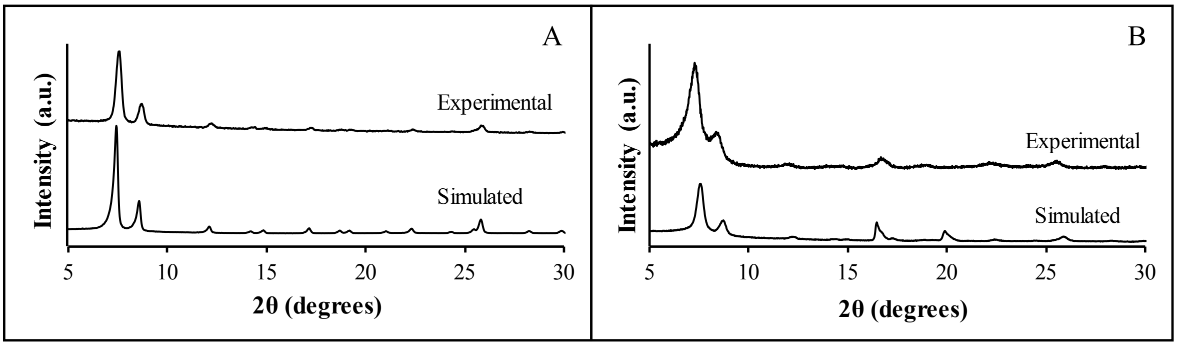

Figure S4. Experimental and simulated XRPD patterns of UiO-66-NH_2 (A) and UiO-66-OH (B).

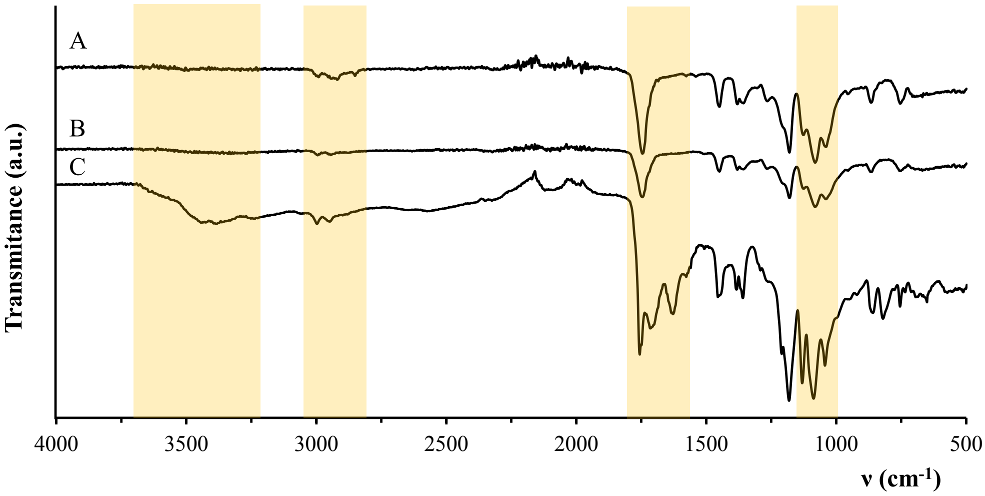

Figure S5. FTIR spectra of 3D-printed scaffolds using neat PLA (A) and PLA-wood composite filaments (B). Trace (C) shows the PLA-wood 3D-printed substrate treated with MA.

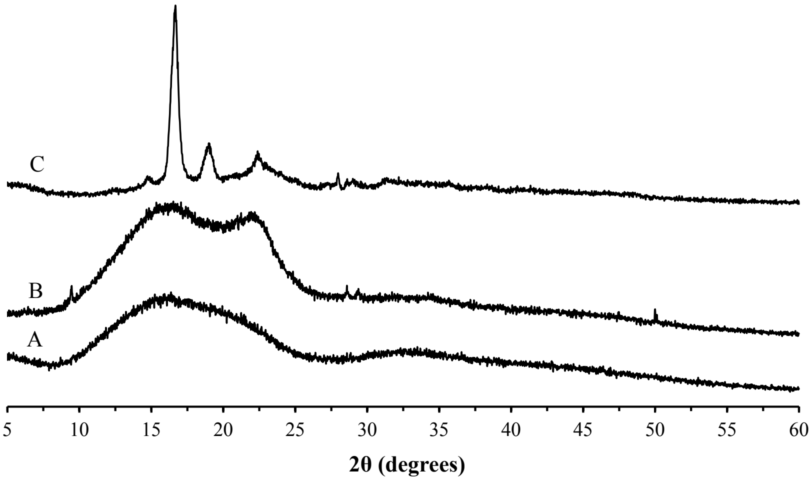

Figure S6. XRPD patterns of 3D-printed scaffolds using neat PLA (A) and PLA-wood composite filaments (B). Trace (C) shows the PLA-wood 3D-printed substrate treated with MA.

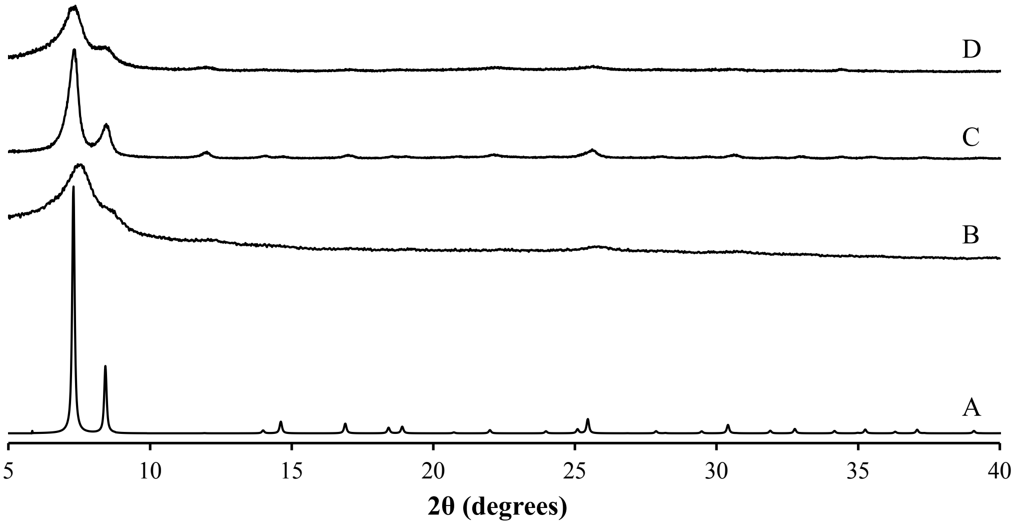

Figure S7. XRPD patterns of UiO-66 obtained in bulk (A) and the UiO-66 crystalline coating removed from 3D-printed surface at different volumes of metal/ligand solution, 5 mL (B), 12 mL (C), and 15 mL (D).

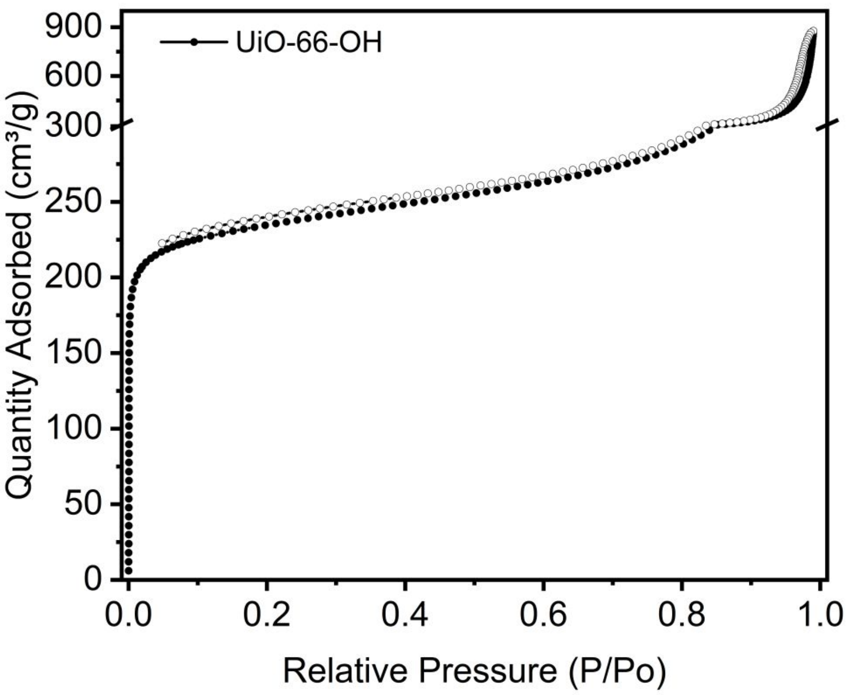

Figure S8. N_2 sorption isotherm at 77K for UiO-66-OH material. Adsorption curve is represented as closed circles, and desorption curve is represented as closed circles.

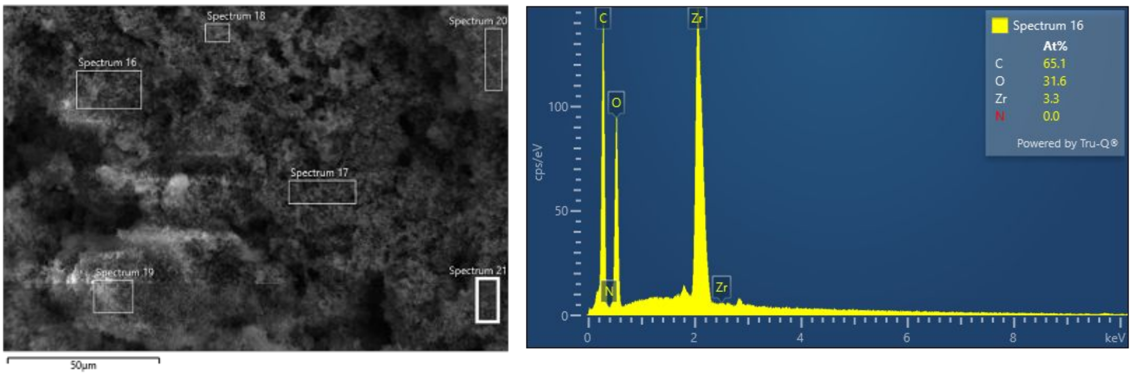

Figure S9. EDS analysis of UiO-66-OH-coated 3D-printed device.

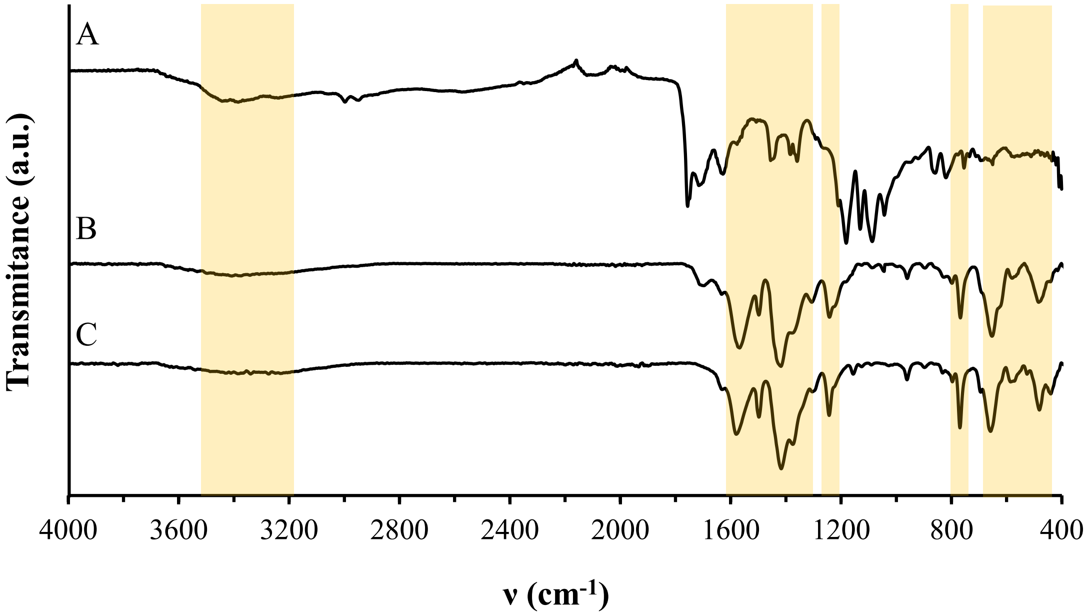

Figure S10. FT-IR spectra of the 3D-printed device before (A) and after coating with the MOF (B) as compared to the as-synthesized UiO-66-OH (C).

Optimization of extraction parameters. The pH of the sample solution plays an important role in the adsorption of these herbicides since it affects both the analyte charge state and the MOF charge surface. Therefore, the effect of this variable on retention efficiency of these analytes was investigated within the pH range of 3.0-7.0. As shown in Fig. S10A, the retention of the pesticides reached the best values at pH 3, and then decreased progressively at higher pHs, this decrease being particularly notable for clopyralid and picloram. These results can be explained as follows. PHs are acidic compounds with low pK_a values between 2.0 and 4.0 (see Table 1), an electrostatic repulsion between these compounds and the negatively charged carboxylate groups of BDC linkers likely would explain the decrease in retention with increasing pH. From these results, pH 3 was selected for further studies.

Salt content in aqueous solutions can change the ionic strength of the solutions or alter the diffusion rate of analytes from aqueous to solid phases.[8, 9] As shown in Fig. S10B, the retention of the analytes increased with the NaCl concentration, rising from 0% to 30% (w/v). This increase was particularly pronounced for clopyralid and picloram, compounds characterized by high water solubility (see Table S1). The addition of salt induced a clear salting-out effect, enhancing the partitioning and retention of these analytes onto the sorbent. Based on these results, a concentration NaCl of 30 % (w/v) was used in the following experiments.

The effect of extraction time was also evaluated within a range of 30 to 120 minutes. As shown in Fig. S10C, herbicide retention reached its maximum at 90 min, with longer extraction times providing no additional improvement in retention. Consequently, an extraction time of 90 minutes was selected for further assays.

The influence of stirring rate (100-400 rpm) on retention of herbicides investigated was investigated (Fig. S10D). When the stirring rate reached 400 rpm, the retention of herbicides remained essentially unchanged, and this rate was selected for the rest of the study.

The selection of an appropriate elution solvent is critical for desorbing the retained herbicides from the MOF@3D-printed device. Additionally, the chosen solvent must be compatible with the stability of the 3D-printed support (see above and Fig. S1). With these considerations in mind, various solvents were tested, including water, MeOH and MeOH-water mixtures at different ratios. Low recoveries were observed when water or MeOH-water mixtures (50:50 v/v) were used as solvents. In contrast, pure MeOH or high MeOH proportions (90:10 v/v) resulted in recoveries ranging from 43% to 65% (Fig. S11). Subsequently, the addition of acid (TFA) to these solvents was tested to enhance elution efficiency of herbicide. As shown, recovery improved with increasing TFA concentrations, which may be attributed to the stronger hydrogen-bond donor behavior of TFA, which reduces the interaction between the analytes and the MOF, thereby facilitating their desorption from the sorbent. The best results (86.8-102%) were obtained using MeOH-water mixtures (90:10 v/v) containing 100 mM TFA.

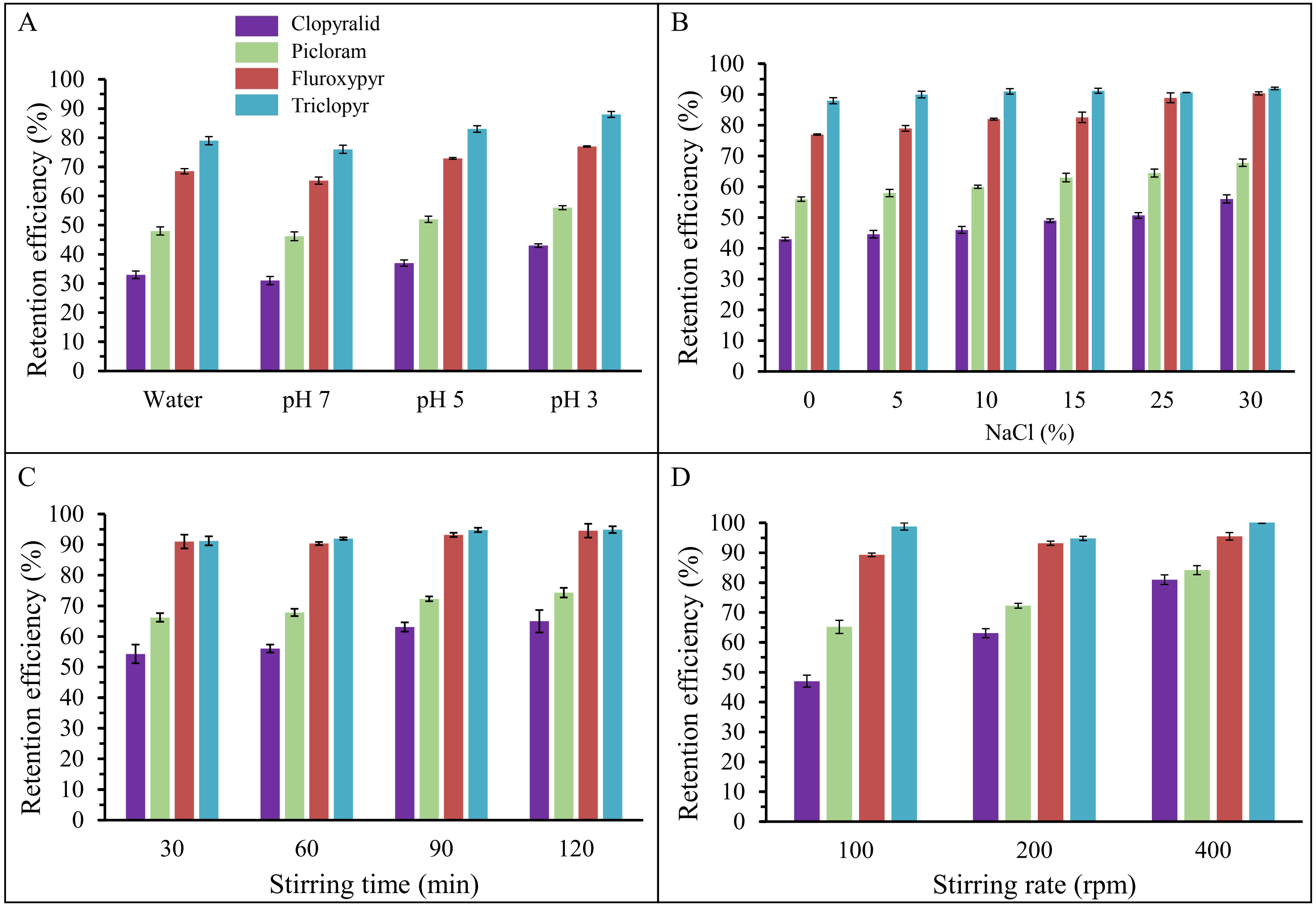

Figure S11. Effect of different variables on the retention of PHs: pH (A), NaCl content (B), stirring time (C) and stirring rate (D).

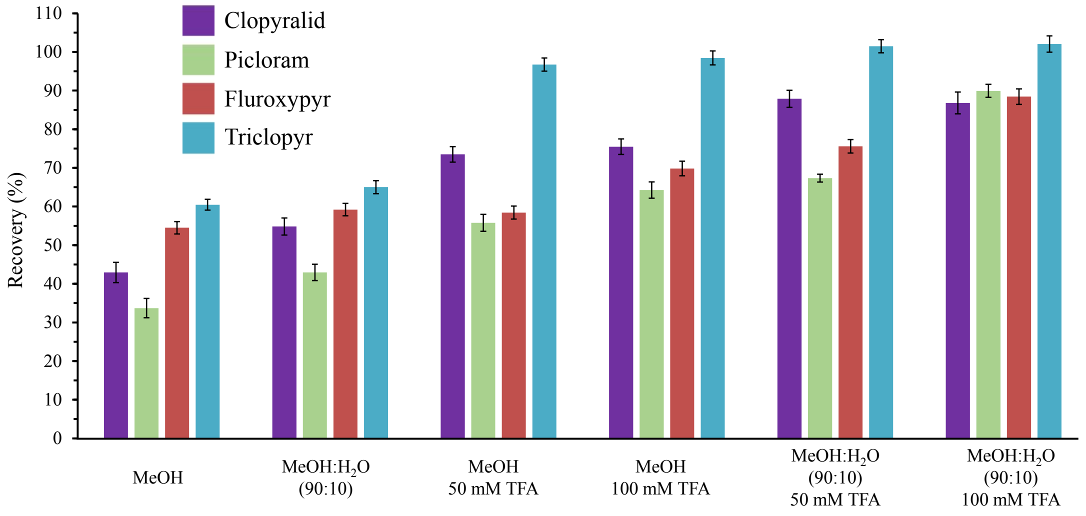

Figure S12. Effect of the desorption solvent on the recovery values of PHs.

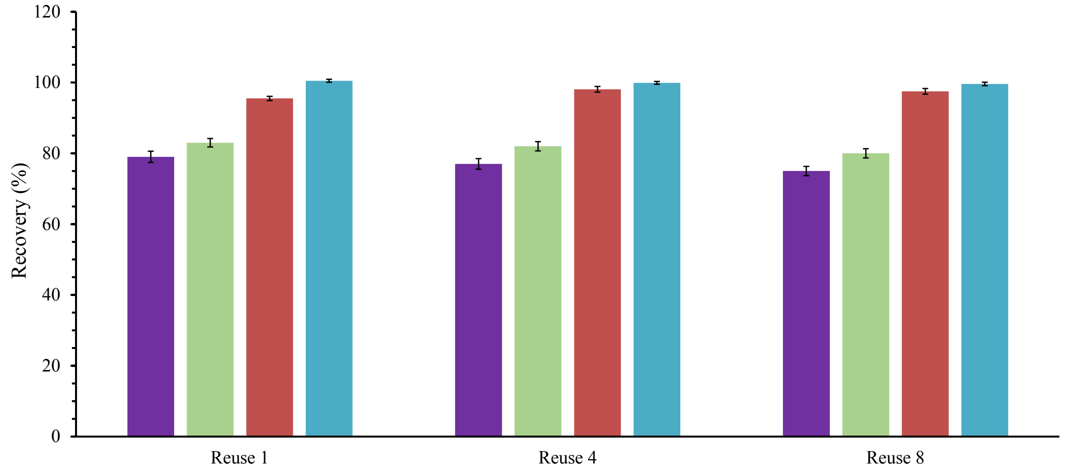

Figure S13. Reusability studies of 3D-printed devices coated with MOF. Colors' code: clopyralid (purple), picloram (green), fluroxypyr (red) and triclopyr (blue).

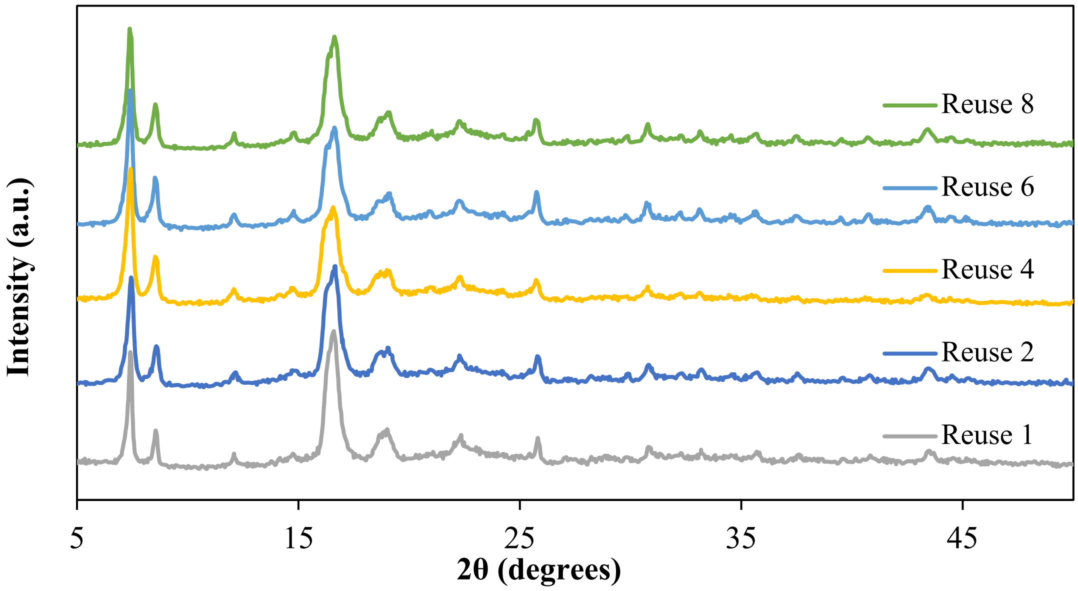

Figure S14. XRPD patterns of the UiO-66-OH@3D-printed device after multiple reuse cycles (reuse 1–8).

Table S5. Comparison of the developed MOF@3D-printed extraction device coupled with HPLC-MS with previously reported methods for PH analysis in water samples.

Analytes	Sample	Method	Material	Recovery (%)	LOD (ng·L^{-1})	Reusability	Ref.
Fluroxypyr and other pesticides	Freshwater	SPE-HPLC-MS/MS	Strata X	42	12.3	-	10
Clopyralid	Drinking water	SPE-HPLC-UV	C18	70-90	20	-	11
Clopyralid, picloram, fluroxypyr, triclopyr and other pesticides	Environmental waters	SPE-HPLC-MS/MS	Oasis® MAX and Bond Elut® Plexa Pax	64-175	6-8	-	12
Clopyralid	Drinking, ground and surface	SPE-HPLC-MS/MS	St-DVB polymer	70-120	9.2-15	-	13
Clopyralid, picloram, triclopyr and other acidic herbicides	Tap water, river water, lake water and pond water	MA-IL-DLLME-UPLC-FLD	Guanidinium IL	78-119	5.7-12.4	-	14
Clopyralid, picloram, fluroxypir, triclopyr	Tap water, water reservoir, seawater and wastewate	Stirred extraction-HPLC-MS/MS	UiO-66-OH@3D-printed device	73-107	1.6-3.2	8	This work

Abbreviations: MA, Magnetic adsorbing assisted; IL-DLLME, Ionic liquid dispersive liquid-liquid microextraction; St-DVB, styrene-divinylbenzene; UPLC, ultra-performance liquid chromatography; FLD, fluorescence detector.

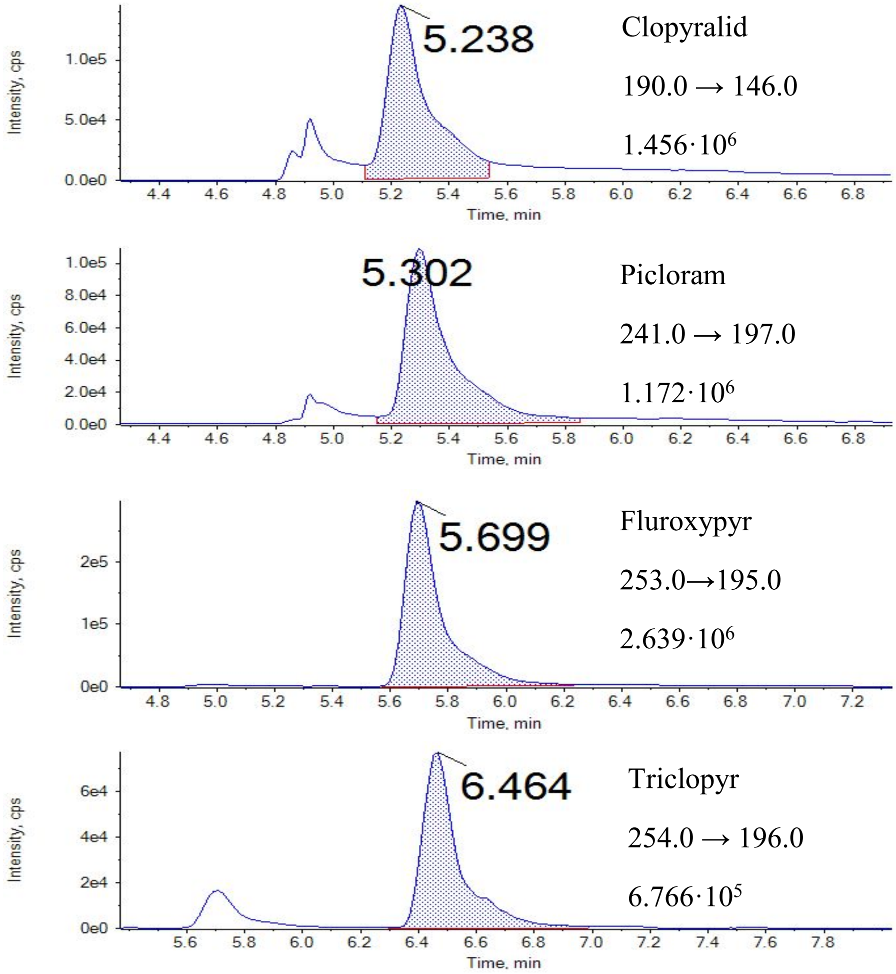

Figure S15. MRM chromatograms of tap water spiked with herbicides at a concentration of 50 µg L^{-1}, after applying the proposed extraction procedure and analyzed under the conditions described in the Experimental Section of the ESM.

References

(1) Pesticide Properties DataBase (PPDB); Agriculture & Environment Research Unit (AERU), University of Hertfordshire. https://sitem.herts.ac.uk/aeru/ppdb/en/atoz_herb.htm (accessed 2026-02-02).

(2) Giménez-Marqués, M.; Cases Díaz, J.; Glatz, J. M.; Mínguez Espallargas, G. MOF-biomolecule biocomposite from hard lewis acid metal cation and uses thereof. EP25383030A.

(3) Srikantamurthy, N. H.; Olorunyomi, J. F.; Doherty, C. M.; Sherrell, P. C.; Mulet, X. Aqueous synthesis of UIO-66 metal-organic frameworks with enhanced crystallinity and surface area. *Adv. Sustainable Syst*. **2025**, *9*, e00854

(4) Ke, F.; Peng, C.; Zhang, T.; Zhang, M.; Zhou, C.; Cai, H.; Zhu, J.; Wan, X. Fumarate-based metal–organic frameworks as a new platform for highly selective removal of fluoride from brick tea. *Sci. Rep.* **2018**, *8*, 939.

(5) Li, C.; Wang, S.; Tao, Z.; Liu, L.; Xu, W.; Gu, X.; Han, Z. Green synthesis of MOF-801(Zr/Ce/Hf) for CO_2/N_2 and CO_2/CH_4 separation. *Inorg. Chem.* **2023**, *62*, 7853–7860.

(6) Guesh, K.; Caiuby, C. A. D.; Mayoral, Á.; Díaz-García, M.; Díaz, I.; Sanchez-Sanchez, M. Sustainable preparation of MIL-100(Fe) and its photocatalytic behavior in the degradation of methyl orange in water. *Cryst. Growth Des.* **2017**, *17*, 1806–1813.

(7) Guan, Y.; Xia, M.; Wang, X.; Cao, W.; Marchetti, A. water-based preparation of nano-sized NH_2-MIL-53(Al) frameworks for enhanced dye removal. *Inorg. Chim. Acta* **2018**, *484*, 180–184.

(8) Li, Y.; Zang, X.; Li, Y.; Zhang, S.; Wang, C.; Wang, Z. Selective extraction of fungicides from fruit samples with defective UIO-66 as solid-phase microextraction fiber coating. *Microchem. J.* **2023**, *190*, 108608.

(9) Pezhhanfar, S.; Farajzadeh, M. A.; Hosseini-Yazdi, S. A.; Mogaddam, M. R. A. The application of Na-Bi MOF as a heterometallic coordination polymer and 2,2-dimethoxypropane as an in-situ-generating ternary solvent for the extraction and preconcentration of some phthalate and adipate esters from tap, well, surface, and river water samples. *Microchem. J.* **2023**, *195*, 109536.

(10) Lazartigues, A.; Fratta, C.; Baudot, R.; Wiest, L.; Feidt, C.; Thomas, M.; Cren-Olivé, C. Multiresidue method for the determination of 13 pesticides in three environmental matrices: Water, sediments and fish muscle. *Talanta*. **2011**, *85*, 1500-1507.

(11) Gu, B.; Meldrum, B.; McCabe, T.; Phillips, S. Enhancing concentration and mass sensitivities for liquid chromatography trace analysis of clopyralid in drinking water. *J. Sep. Science.* **2012**, *35*, 185-192.

(12) Zhang, P.; Bui, A.; Rose, G.; Allinson, G. Mixed-mode solid-phase extraction coupled with liquid chromatography tandem mass spectrometry to determine phenoxy acid, sulfonylurea, triazine and other selected herbicides at nanogram per litre levels in environmental waters. *J. Chromatogr. A* **2013**, *1325*, 56–64.

(13) Mann, O.; Pock, E.; Wruss, K.; Wruss, W.; Krska, R. Development and validation of a fully automated online-SPE–ESI–LC–MS/MS multi-residue method for the determination of different classes of pesticides in drinking, ground and surface water. *Int. J. Environ. Anal. Chem.* **2016**, *96*, 353–372.

(14) Fan, C.; Yang, J.; Liang, Y.; Dong, H.; Zhang, W.; Tang, G.; Cao, Y. Effective tuning guanidinium ionic liquid as greener solvent for fast and sensitive determination of auxin herbicides. *Microchem. J.* **2019**, *144*, 73-82.
